# DELLY: structural variant discovery by integrated paired-end and split-read analysis

**DOI:** 10.1093/bioinformatics/bts378

**Published:** 2012-09-03

**Authors:** Tobias Rausch, Thomas Zichner, Andreas Schlattl, Adrian M. Stütz, Vladimir Benes, Jan O. Korbel

**Affiliations:** ^1^ European Molecular Biology Laboratory (EMBL), Genome Biology, Meyerhofstr. 1, 69117 Heidelberg, Germany; ^2^EMBL European Bioinformatics Institute, Wellcome Trust Genome Campus, Hinxton, Cambridge CB10 1SD, UK; ^3^European Molecular Biology Laboratory (EMBL), Core Facilities and Services, Meyerhofstr. 1, 69117 Heidelberg, Germany

## Abstract

**Motivation:** The discovery of genomic structural variants (SVs) at high sensitivity and specificity is an essential requirement for characterizing naturally occurring variation and for understanding pathological somatic rearrangements in personal genome sequencing data. Of particular interest are integrated methods that accurately identify simple and complex rearrangements in heterogeneous sequencing datasets at single-nucleotide resolution, as an optimal basis for investigating the formation mechanisms and functional consequences of SVs.

**Results:** We have developed an SV discovery method, called DELLY, that integrates short insert paired-ends, long-range mate-pairs and split-read alignments to accurately delineate genomic rearrangements at single-nucleotide resolution. DELLY is suitable for detecting copy-number variable deletion and tandem duplication events as well as balanced rearrangements such as inversions or reciprocal translocations. DELLY, thus, enables to ascertain the full spectrum of genomic rearrangements, including complex events. On simulated data, DELLY compares favorably to other SV prediction methods across a wide range of sequencing parameters. On real data, DELLY reliably uncovers SVs from the 1000 Genomes Project and cancer genomes, and validation experiments of randomly selected deletion loci show a high specificity.

**Availability:** DELLY is available at www.korbel.embl.de/software.html

**Contact:** tobias.rausch@embl.de

## 1 INTRODUCTION

Genomic structural variants (SVs), including gains and losses of DNA segments and balanced rearrangements, are a major form of variation in the human genome (Conrad *et al.*, 2010; Mills *et al.*, 2011; Sudmant *et al.*, 2010). Polymorphic SVs are a major contributor to common traits, including common diseases (McCarroll *et al.*, 2008; Stranger *et al.*, 2007) whereas rare SVs present in the germline are the cause of many rare genomic disorders (Korbel *et al.*, 2009b; Zhang *et al.*, 2009). Furthermore, somatic structural rearrangements, often highly complex, play a pivotal role in the development of aggressive cancers (Rausch *et al.*, 2012; Stephens *et al.*, 2011). A critical first step in associating SVs with phenotypes is the discovery and precise mapping of these DNA sequence variants.

The introduction of massively parallel sequencing (MPS) technologies has led to considerable advances in the discovery and genotyping of structural variants in the germline and in somatic cells (Campbell *et al.*, 2008; Korbel *et al.*, 2007; Sudmant *et al.*, 2010). Several complementary approaches have been developed to leverage MPS data for SV discovery. These include methods using the analysis of abnormally mapping pairs of MPS fragments, so-called paired-end mapping methods (Chen *et al.*, 2009; Korbel *et al.*, 2007, 2009a), read-depth analysis that detects SVs by analyzing the read depth-of-coverage (Abyzov *et al.*, 2011; Campbell *et al.*, 2008; Chiang *et al.*, 2009), split-read analysis that evaluates gapped sequence alignments to detect SVs (Abyzov and Gerstein, 2011; Wang *et al.*, 2011; Ye *et al.*, 2009) and sequence assembly that enables the discovery of novel sequence insertions (Hajirasouliha *et al.*, 2010). A recent survey of SVs in 185 human genomes by the 1000 Genomes Project's (1000GP) SV Analysis Group reported over 28 000 SVs based on these four SV discovery approaches in the 1000GP pilot phase (Mills *et al.*, 2011). Paired-end mapping made the comparably largest contribution to this list of reported SVs. However, only approaches that combined two complementary signatures for SV discovery by integrating paired-end mapping and read-depth analysis met the prespecified specificity threshold of a false-discovery rate (FDR) ≤10% (Handsaker *et al.*, 2011; Mills *et al.*, 2011).

Recently, the parameters at which genomes are sequenced by MPS have considerably changed. Although most reads in the 1000GP pilot phase had a length of ≈36 bp, the average read length used in the project's main phase and in current cancer genome projects has increased by 3-fold (≈105 bp). At the same time, the number of reads is reduced by 3-fold to yield comparable sequence coverage. Furthermore, several large-scale genome studies have begun to generate paired-end sequencing libraries with two different insert sizes (Pleasance *et al.*, 2010; Rausch *et al.*, 2012). One library is usually smaller than 500 bp, and the other, commonly referred to as a mate-pair library, is typically larger than 2 kb and up to 5 kb, to facilitate sensitive SV detection across a widened SV size-spectrum and in repetitive areas of the genome. These changes in sequencing parameters and strategy significantly affect SV discovery. Split-read analysis methods should benefit from relatively long reads whereas read counting methods are expected to suffer from a 3-fold reduction in the number of sequenced reads. Compared to read-depth and paired-end analysis, split-read analysis has so far been limited to the detection of small SVs (Ye *et al.*, 2009) as well as SVs in ‘unique’ genomic regions, devoid of repeats and segmental duplications (Wang *et al.*, 2011). Most importantly, certain classes of SVs, including tandem duplications, inversions, translocations and particularly highly complex events, are not yet sensitively ascertained in split-read-based analyses.

**Fig. 1.**
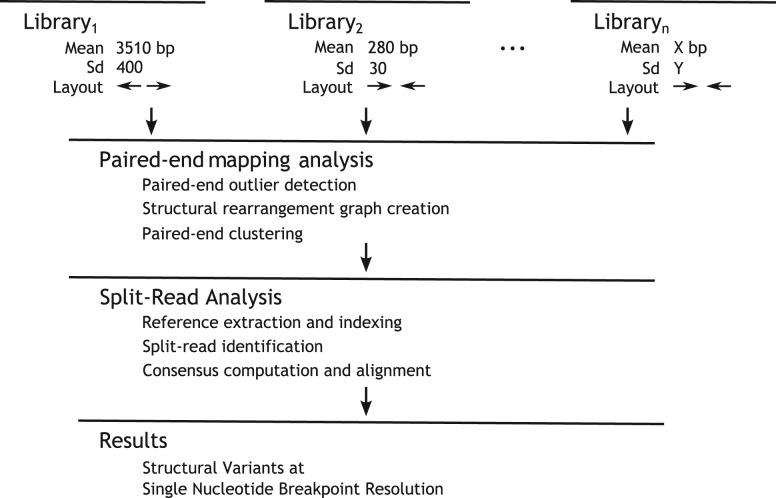
DELLY design: short-range and long-range paired-end libraries are analyzed for discordantly mapped read pairs. Paired-end predicted structural variants are then refined using split-reads and reported at single-nucleotide breakpoint resolution

Herein, we report a new integrative approach, called DELLY, that combines short-range and long-range paired-end mapping and split-read analysis for the discovery—at single nucleotide resolution—of balanced and unbalanced forms of structural variation, i.e. deletions, tandem duplications, inversions and translocations, achieving high sensitivity and specificity throughout the genome and for SVs falling into a wide size spectrum. Thereby, DELLY has been specifically geared towards enabling SV calling in the presence of different paired-end sequencing libraries with distinct insert sizes (Fig. 1). To our knowledge, no single SV detection approach presently offers such integrative SV calling at nucleotide resolution in MPS data, which is of relevance for assessing the origin and functional impact of SVs in individual genome sequencing efforts focused on germline or somatic genome variation.

## 2 METHODS

The input of DELLY is a set of aligned MPS reads in SAM/BAM format (Barnett *et al.*, 2011; Li *et al.*, 2009), where each input file is assumed to be a separate library with a distinct insert size median and standard deviation. All input libraries are analyzed jointly to achieve optimal sensitivity. The method consists of two separate components, a paired-end mapping analysis component and a split-read analysis component (Fig. 1).

### 2.1 Paired-end mapping analysis

**Fig. 2.**
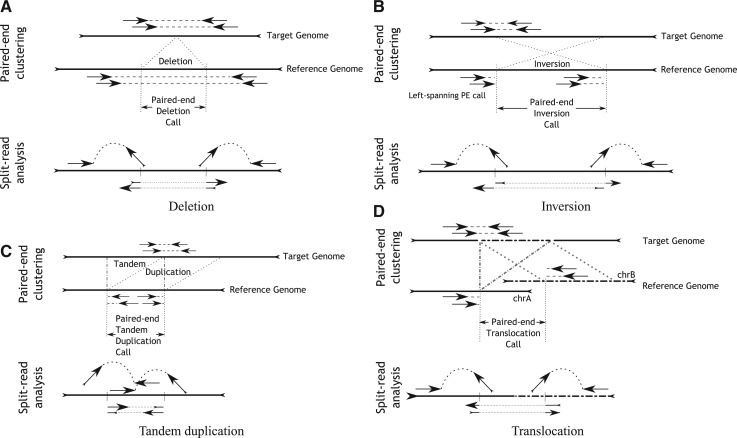
Paired-end clustering and split-read detection for a deletion (A), inversion (B), tandem duplication (C) and translocation (D)

For each input BAM file, DELLY computes the default read-pair orientation and the paired-end insert size distribution characterized by the median and standard deviation of the library. Based on these parameters, DELLY then identifies all discordantly mapped read-pairs that either have an abnormal orientation or an insert size greater than the expected range. DELLY hereby focuses on uniquely mapping paired-ends and the default insert size cutoff is three standard deviations from the median insert size. All SV types induce a characteristic mapping pattern (Fig. 2) that is leveraged by DELLY in the following way:

- ‘Deletions’ are detected as paired-end outliers at the far end of the insert size distribution but at default library orientation.
- ‘Inversions’are detected as abnormally oriented paired-ends where an orientation change of one read leads to the default library orientation. Left-spanning and right-spanning paired-ends are differentiated as outlined in Figure 2.
- ‘Tandem duplications’ are detected as paired-ends where the first and second read changed their relative order but kept the alignment strand induced by the default library orientation.
- ‘Translocations’ are detected as paired-ends mapping to different chromosomes. Four possible types of translocations are differentiated, whether the two chromosomes are in sorted order and whether the two chromosomes are inverted relative to each other (Fig. 4).

**Fig. 3.**
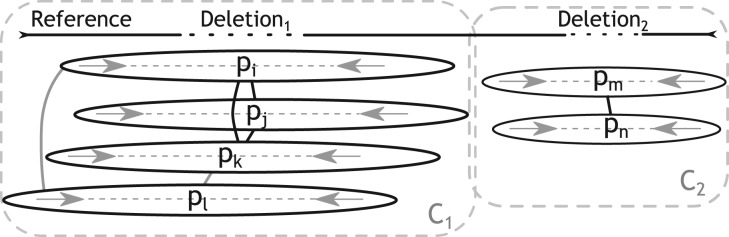
Graph-based paired-end clustering: a graph *G* of structural rearrangements with two connected components *C*_1_ and *C*_2_ and two maximal cliques (*p_i_*,*p_j_,p_k_*) and (*p_m_*,*p_n_*). The non-clique edges are in gray. For simplicity, edge weights have been omitted

All discordantly mapped paired-ends are binned by chromosome and sorted according to the left-most alignment position. For translocations, DELLY sorts all paired ends according to the lexicographically smaller chromosome. This sorted vector of discordantly mapped paired ends is subsequently used to build an undirected, weighted graph *G*(*V*,*E*) that indicates which paired-ends support the same structural rearrangement. For each paired-end *p_i_*, the graph *G* contains one node *v_i_* ∈*V*. An edge *e_v_i__*,*_v_j__* = {*v_i_v_j_*}∈*E* indicates that both paired ends support the same SV. This demands that *p_i_* and *p_j_* have the same orientation (change) with respect to their library orientation and that the absolute difference between the left and right ends of *p_i_* and *p_j_* are within the expected insert size range. The weight of edge *e_v_i_v_j__*, denoted as *w*(*e_v_i_v_j__*), is the absolute difference between the predicted SV sizes induced by the mapping locations of the paired-ends *p_i_* and *p_j_*, respectively. Since this is not possible for translocations, DELLY takes in this case the sum of the absolute differences in the left-most alignment position of both read-pairs. To achieve maximum specificity, we only cluster paired-ends that show the same mapping pattern. In particular, we cluster left- and right-spanning paired-ends separately for inversions and we cluster the four types of translocations separately. The algorithm to build the graph is a simple line-sweep algorithm with worst-case running time *O*(*n*^2^), where *n* is the number of discordantly mapped paired-ends for a given chromosome. However, we only need to traverse the sorted vector from a given paired-end *p_i_* until we reach the first *p_j_*, where the distance between the first read of *p_i_* and *p_j_* is greater than the expected range. Hence, in practice, the graph *G*(*V*,*E*) can be build very fast, an example graph is shown in Figure 3.

**Fig. 4.**
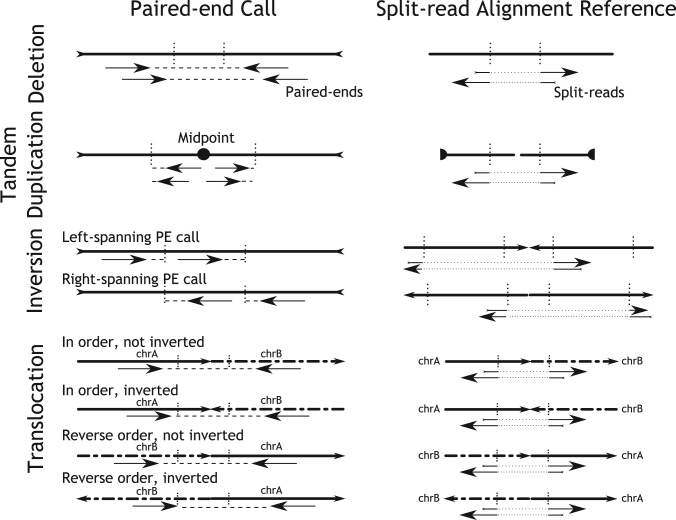
The build-up of the split-read alignment reference depends on the type of paired-end call. For tandem duplications, inversions and translocations, we modify the reference in such a way that a standard ‘deletion-type’ split-read alignment can be carried out

Assuming ideal conditions, the graph *G* contains one fully connected component for each structural rearrangement. Each variant could thus be identified by computing the connected components *C_i_* of the graph. Due to inadequate fragment shearing, sequencing errors, ambiguous read mapping locations and incomplete reference sequences, most components *C_i_* are not fully connected. In other words, the subgraph induced by the component, denoted as *G_C_i__* (*V_C_i__*, *E_C_i__*), is not a clique. We do not discard such components but rather sort the edges of each component by weight and identify a maximal clique *M_i_* heuristically in the component *C_i_* using the edge of smallest weight as the seed of the clique.

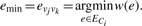

We then extend this clique *M_i_* = {*v_j_*, *v_k_* } from the seed-edge by means of searching for the next best edge *e_s_* such that

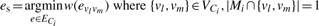

and requiring that the subgraph induced by *M_i_* ∪ {*v_l_*, *v_m_*} is a clique. If no such edge *e*_s_ exists, the clique is maximal for the seed-edge *e*_min_ and reported as the paired-end cluster of size |*M_i_*| for this SV. This procedure by definition implies that singleton nodes in *G* are discarded. The maximal clique *M_i_* is also used to estimate the start and end coordinate of the SV. In case of a deletion, for instance, the start and end position is estimated as the maximal begin position of all paired-ends of the cluster and the minimal end position of all paired-ends of the cluster, respectively.

Each rearrangement type is analyzed separately and consequently, deletions, inversions, tandem duplications and translocations can be overlapping or nested. For rearrangements of the same type that share a common beginning or end (such as two deletions *d*_1_[*a,b*] and *d*_2_[*a,c*]) the method currently identifies only a single event, where a possible extension of the method would be a full enumeration of all maximal cliques in each connected component.

## 2.2 Split-read analysis

The paired-end clusters identified in the previous mapping analysis are interpreted as breakpoint-containing genomic intervals, which are subsequently screened for split-read support to fine map the genomic rearrangements at single-nucleotide resolution and to investigate the breakpoints for potential microhomologies and microinsertions. The split-read analysis is a multi-step process, including (i) candidate split-read search, (ii) SV reference extraction, (iii) indexing and *k*-mer counting, (iv) detecting the best supported breakpoint, (v) split-read consensus computation and (vi) an alignment of the split-read consensus sequence to the SV reference region.

For each putative SV interval *SV_i_*, DELLY searches for the local presence of single-anchored paired ends. A single-anchored paired-end is a read pair where one read maps to the reference and the other read is unmapped. The non-mapped read is a likely candidate for a split-read (Fig. 2). Optionally, DELLY can also use all mapped reads in the breakpoint region to take into account soft-clipped reads or reads mapped with ‘sloppy’ ends. To gain maximum specificity, DELLY records and later enforces for each non-mapped read one alignment direction (forward or reverse strand), which can be inferred from the mapped read and the default library orientation. This default orientation can also be used to infer whether a structural variant breakpoint of a mapped single-anchored read should be expected to the right or to the left of the given mapped read. The split-reads are collected efficiently using the following algorithm:

1. Sort all SV start and end breakpoints by chromosome and position.
2. Search bam files once for single-anchored reads (or optionally all reads).
3. For each read, use the mapped partner and the default library orientation to determine the search direction (increasing/decreasing genomic coordinates) and binary search to detect the closest SV breakpoint.
4. If a read maps within two standard deviations of a breakpoint, assign this read to the set of putative split-reads *R*_SV_*i*__ where *i* ∈ 1,...,*m* and *m* is the number of paired-end called SVs.

In centro- and telomeric regions, we frequently observed huge pile-ups of reads and many SV predictions, indicative of extensive inter-individual variability and possibly unfinished reference genome sequence assemblies present in these repeat-rich regions. This led to thousands of putative split-reads for some SV calls that would be prohibitively expensive to align. However, we also did not intend to a priori exclude such regions, some of which are known SV hotspots (Mills *et al.*, 2011) and of clinical importance. As a result, we decided to limit the maximum number of split-reads per SV interval to |*R*_SV_*i*__ | ≤ *L*, where the default for *L* is 1000.

For deletions, the build-up of the split-read alignment reference demands a simple extraction of the paired-end SV interval from the genome. The prefix and suffix alignments of a split-read are by definition in the same orientation and in the expected order for deletions. For inversions, tandem duplications and translocations, a direct alignment to the reference would demand either a change in the orientation (inversions) or a change in the prefix–suffix order (tandem duplications) or potentially both changes for translocations (Fig. 2). To simplify the subsequent split-read alignment, we decided to modify the SV reference depending on the paired-end SV call to then carry out a standard ‘deletion-type’ split-read search for all SV types, as shown in Figure 4 for the different classes of paired-end SV calls.

**Fig. 5.**
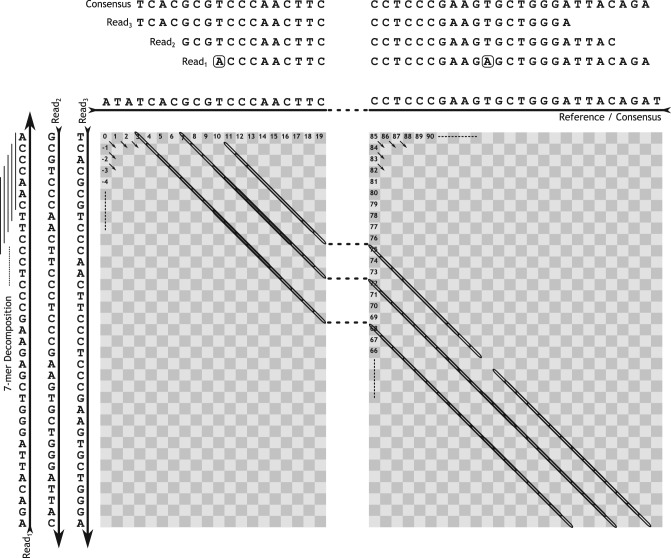
Using an index of the SV reference, DELLY records the number of seven-mer hits per diagonal for each read. In the above example, Read_1_ induces three hits on diagonal 10 and 12 on diagonal 75. Read_2_ induces seven hits on diagonal 7 and 16 hits on diagonal 72. Read_3_ induces 11 hits on diagonal 3 and 12 hits on diagonal 68. For all the reads the offset between the two most supported diagonals is 65 bp suggesting an SV length of 65 bp. The consensus sequence of the three reads is shown at the top

A split-read alignment by dynamic programming is prohibitively expensive for the full set of putative split-reads and hence, DELLY uses a fast *k*-mer-based filtering technique to identify candidate split-reads. The default *k*-mer-index of the SV containing reference region for SV*_i_* uses *k* = 7. *k* adjusts the sensitivity and specificity of DELLY's split-read search. Simulated SVs showed that a small value of *k* provides the best recall, in particular for short reads (≈36 bp) and low coverage (≤5×). Due to the small, paired-end guided reference region, specificity remained high even for small *k*. Given this index for SV*_i_*, DELLY now maps each *k*-mer of a read *r* ∈*R*_SV_*i*__against the index and normalizes each *k*-mer hit by the offset of the *k*-mer in the read. Thus, in terms of the alignment matrix of a given read against the SV region we count the *k*-mer hits by alignment diagonal (Fig. 5). To increase specificity, DELLY only applies this *k*-mer counting to one alignment direction, forward or reverse strand, depending on the paired-end induced alignment direction. During this *k*-mer counting, we ignore any *N* s in the read or the reference. Any sequencing error can destroy up to *k k*-mer hits. For instance, the second boxed ‘A’ in Figure 5 causes a loss of seven k-mer hits. Due to repeats, a given read *k*-mer can have multiple hits in the SV region. Because of that DELLY post-processes the diagonals in decreasing order according to the number of *k*-mer hits. In this procedure, each read *k*-mer is flagged as ‘used’ once one of its diagonals has been processed. This ensures that every read *k*-mer is assigned only once to the best supported diagonal. Finally, we discard all diagonals that have less than *k*_min_ hits, the default for *k*_min_ is 3. If in the end a read *r* ∈R_SV_*i*__ has less than two diagonals above this threshold the read *r* is discarded from the set of putative split-reads *R*_SV_*i*__ We further require that the two diagonals with the most hits account in total for at least half of the *k*-mers of the read, otherwise the read is also discarded. This implies that any non-template reference insertion (see below) can only be found if it is smaller than half the read-length. All candidate split-reads with at least two diagonals above the *k*-mer hit threshold are further processed by means of sorting the diagonals of a given read by their diagonal index and recording the offset between two consecutive diagonals. This offset between consecutive diagonals corresponds for a true SV event to the size of the SV. For instance, in the small example shown in Figure 5 all three reads support an offset of 65 bp.

This single read *k*-mer search is still unbiased and not guided by the paired-end predicted SV length to avoid false positives. Only after having collected all predicted offsets from all putative split-reads, we extract the maximum supported offset for each structural variant SV*_i_*. By default, DELLY requires at least two split-reads but this value can be increased on the command-line to enforce specificity. We then take the subset of reads *R*_SV_*i*__^′^⊆*R*_SV_*i*__ that supported this offset and build the consensus sequence *c* = *c*_1_*c*_2_ ···*c_n_* of length *n*, where we apply a simple majority vote in each consensus column (Fig. 5)

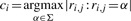

where ∑ is the set of nucleotides {*A*,*C*,*G*,*T*}, *c_i_* is the consensus nucleotide for column *i* and *j* is the read index (or row index in the consensus alignment). At the moment, DELLY constructs only a gapless consensus sequence, which can be extended in the future using a realignment method (Anson and Myers, 1997) that accounts for insertion and deletion sequencing errors.

The final consensus sequence *c* is now aligned to the reference region of SV*_i_* using a sensitive double dynamic programming. DELLY computes two scoring matrices *S_F_* and *S_R_*, one for the forward and one for the reverse alignment using the Gotoh algorithm with affine gap penalties from the SeqAn library (Döring *et al.*, 2008; Rausch *et al.*, 2008). Both scoring matrices are then used to define the optimal split, allowing for potential non-template insertions. This method resembles an algorithm proposed for assembled contig alignment to a reference genome, called AGE (Abyzov and Gerstein, 2011). DELLY follows the AGE approach that allows microinsertions at the breakpoint with two important changes. First, DELLY uses affine gap penalties to avoid alignment regions interspersed with gaps and second, DELLY uses a global instead of a local alignment algorithm since the consensus sequence was derived from the read data and should thus be alignable across the full length. In brief, the forward scoring matrix *S_F_* is used to compute a forward scoring vector *f* = (*f*_1_,*f*_2_,···, *f_n_*). Element *f_i_* is the maximum score of the best prefix consensus alignment *c*_1_*c*_2_ ···*c_i_* to the reference. Likewise, a reverse scoring vector *r* = (*r*_1_,*r*_2_,···, *r_n_*) is computed from the reverse scoring alignment matrix *S_R_* where *r_j_* is the maximum score of the best suffix consensus alignment *c_n_c_n_*_−1_ ···*c_j_* to the reverse reference. The optimal ‘left’ and ‘right’ breakpoint of the structural variant in the read is then defined as the maximum total alignment score using the two scoring vectors *f* and *r*.

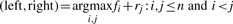

We do not require *j* =*i*+1 to accommodate non-template microinsertions at the breakpoint, which are thought to be commonly introduced by DNA repair mechanisms. The exact breakpoint in the reference can be calculated from the scoring matrices *S_F_* and *S_R_*, the maximum alignment score giving rise to *f_i_* and *r_j_* and translating back the coordinates from the ‘deletion-type’ split-read alignment reference to the original SV region. In a final step, DELLY checks whether the split-read predicted SV length confirms the paired-end estimated SV length allowing for a difference of up to *c* percent in length, with *c*=10% as the default.

### 2.3 Call annotation and call merging

Paired-end calls are annotated by the number of supporting pairs and their average mapping quality. Split-read refined calls are annotated by the number of split-reads and the split-read consensus alignment quality against the reference. For inversions and translocated segments, DELLY is able to merge the left- and right-spanning paired-end or split-read calls. For paired-end inversion calls, DELLY merges only complementing left- and right-spanning calls that have a reciprocal overlap of at least 80%. If multiple calls could be merged, DELLY takes a best first approach, by merging those calls first that have the smallest absolute distance in their predicted breakpoint position. For translocated segments, DELLY verifies that both paired-end/split-read calls support the same relative orientation (Fig. 4) and that the predicted breakpoints on the chromosome where the segment is inserted are less than *z* bp apart, where *z* is by default 300 bp. If there are multiple translocation calls that could be merged, DELLY first merges calls where the absolute difference in the predicted insertion position is minimal.

## 3 RESULTS

### 3.1 Benchmarking DELLY on simulated data

DELLY was benchmarked on simulated data to estimate the sensitivity (S) and the positive-predictive value (PPV) under different sequencing parameter settings, and to compare DELLY to other structural variant calling algorithms that are suitable for detecting SVs in the germline and in somatic tissues, namely Pindel version 0.2.4 (Ye *et al.*, 2009), Breakdancer version 1.1 (Chen *et al.*, 2009), GASV version 1.4 (Sindi *et al.*, 2009) and HYDRA version 0.5.3 (Quinlan *et al.*, 2010). The source sequence was derived from a randomly selected 10 Mbp region of *chr16* from the latest human genome reference (hg*19*). SVs were randomly simulated and integrated into the source sequence in a non-overlapping manner. The wgsim (Li *et al.*, 2009) read simulator was used to sample reads from the modified source sequence assuming a 1% sequencing error rate under different insert size, coverage and read length assumptions (Table 1). All simulated reads were mapped to the original 10 Mbp source sequence using the Burrows-Wheeler Aligner (BWA) (Li *et al.*, 2008) with default parameters. For translocations, we simulated translocated segments, where random regions of the 10 Mbp source sequence were extracted and removed after the read simulation and then added as separate chromosomes during alignment. The final BWA alignment file was used for calling structural variants. The predicted SVs were then compared to the simulated events and each of these simulations was repeated five times for each fixed parameter setting, where the median results across these five simulations are reported and summarized in Table 1. In paired-end mode, DELLY (PE) recovers more than 90% of the simulated SVs on almost all tested settings. Integrating paired-end and split-read mapping, denoted as DELLY (SR), achieves a very high-positive predictive value, unmet by most of the other methods, at the cost of only a slight decrease in sensitivity compared to DELLY (PE). The sensitivity of DELLY (SR) depends on the coverage and read-length, suggesting that for short-reads and low coverage genomes paired-end methods are a better choice, with Breakdancer offering the best trade-off between sensitivity and specificity. Given a sufficient coverage, Pindel is a very good choice for inversions at the size range where it operates (1–10 kbp). Across all SV types, the performance of DELLY is robust across the simulated sequencing parameter space.

**Table 1.** Sensitivity and positive predictive value of the methods to predict 100 non-overlapping, randomly simulated deletions, tandem duplications and inversions of length 500 bp ≤ *x* ≤ 5000 bp in a target sequence of 10 Mbp that was randomly sampled from *chr16* of the *hg19* human genome reference

	Coverage				Insert size, *N* (*μ*,*σ* = 0.1·*μ*)			Insert size SD, *N* (300,*σ*)			Read length (bp)		
Tool	5	10	15	30	*N* (200,20)	*N* (400,40)	*N* (600,60)	*N* (300,10)	*N* (300,40)	*N* (300,70)	35	55	105
**Deletions**															
DELLY (PE)	**0.95**/0.95	**0.99**/0.85	**0.98**/0.67	**1.00**/0.42	**0.97**/0.89	**1.00**/0.64	**1.00**/0.49	**0.99**/0.79	**1.00**/0.69	**0.98**/0.56	**1.00**/0.32	**1.00**/0.54	**0.98**/0.88
DELLY (SR)	0.74/**1.00**	0.96/0.99	**0.98**/0.98	**1.00**/**1.00**	**0.97**/**1.00**	0.99/**1.00**	0.96/**0.98**	0.98/0.99	0.99/**1.00**	**0.98**/0.99	0.86/0.99	0.99/**1.00**	**0.98**/**1.00**
Pindel	0.45/**1.00**	0.85/0.99	0.94/0.95	0.99/0.93	0.92/0.98	0.95/0.97	0.91/0.87	0.94/0.99	0.95/0.96	0.90/0.94	0.72/0.89	0.93/0.99	0.93/0.95
Breakdancer	0.89/**1.00**	0.97/**1.00**	0.94/**1.00**	0.98/0.99	0.88/**1.00**	0.96/**1.00**	0.88/0.97	0.97/**1.00**	0.99/**1.00**	0.94/**1.00**	0.97/**1.00**	0.98/**1.00**	0.94/**1.00**
GASV	0.44/0.01	0.44/0.01	0.39/0.03	0.28/0.12	0.51/0.01	0.31/0.07	0.13/0.04	0.46/0.02	0.41/0.03	0.36/0.04	0.23/0.08	0.36/0.07	0.46/0.01
HYDRA	0.34/0.77	0.46/0.73	0.52/0.72	0.68/0.86	0.49/0.80	0.49/0.60	0.34/0.26	0.47/0.65	0.56/0.84	0.55/0.57	0.47/0.15	0.54/0.47	0.46/0.88
**Tandem Dupl.**															
DELLY (PE)	**0.96**/0.99	**0.96**/0.91	**1.00**/0.96	**1.00**/0.99	**0.88**/0.93	**1.00**/0.99	**1.00**/0.97	**0.98**/0.92	**1.00**/0.99	**0.96**/0.76	**0.99**/0.94	**0.99**/0.88	**0.99**/0.99
DELLY (SR)	0.71/**1.00**	0.89/0.99	0.97/0.99	**1.00**/**1.00**	0.87/0.99	**1.00**/**1.00**	0.99/0.99	0.94/0.95	**1.00**/**1.00**	0.94/0.94	0.86/**1.00**	0.96/0.99	0.97/0.99
Pindel	0.49/**1.00**	0.73/**1.00**	0.95/**1.00**	0.99/0.99	0.85/**1.00**	0.93/0.99	0.97/0.99	0.84/**0.99**	0.97/**1.00**	0.84/**0.99**	0.76/**1.00**	0.87/**1.00**	0.97/**1.00**
Breakdancer	0.93/0.99	0.83/**1.00**	0.95/0.99	0.99/**1.00**	0.77/**1.00**	0.97/0.98	0.98/**1.00**	0.85/**0.99**	0.99/**1.00**	0.83/0.97	0.97/**1.00**	0.93/**1.00**	0.95/**1.00**
GASV	0.51/0.52	0.38/0.44	0.39/0.39	0.31/0.31	0.40/0.47	0.30/0.31	0.13/0.15	0.41/0.46	0.43/0.43	0.36/0.41	0.27/0.27	0.29/0.31	0.50/0.51
HYDRA	0.26/0.79	0.32/0.43	0.30/0.46	0.33/0.52	0.33/0.72	0.19/0.32	0.12/0.16	0.22/0.37	0.29/0.59	0.29/0.37	0.32/0.12	0.27/0.28	0.30/0.75
**Inversions**															
DELLY (PE)	**1.00**/**1.00**	**0.99**/0.97	**1.00**/0.94	**1.00**/0.96	**0.99**/0.97	**1.00**/0.97	**1.00**/0.93	**1.00**/0.93	**1.00**/0.96	**1.00**/0.97	**1.00**/0.79	**1.00**/0.91	**1.00**/**1.00**
DELLY (SR)	0.62/**1.00**	0.82/0.96	0.89/0.98	0.92/**1.00**	0.90/**1.00**	0.92/**1.00**	0.90/0.98	0.91/0.95	0.91/0.99	0.92/**0.99**	0.68/**1.00**	0.92/0.98	0.85/0.99
Pindel	0.87/**1.00**	0.97/**1.00**	0.97/**0.99**	0.99/0.97	0.97/**1.00**	0.99/0.99	0.98/**0.99**	0.99/**1.00**	0.98/**1.00**	0.99/**0.99**	0.91/0.98	0.98/**1.00**	**1.00**/**1.00**
Breakdancer	0.86/0.67	0.80/0.78	0.83/0.86	0.75/0.76	0.95/0.55	0.69/0.69	0.43/0.43	0.84/0.84	0.79/0.81	0.75/0.75	0.76/0.77	0.78/0.78	0.98/0.51
GASV	0.81/0.80	0.73/0.71	0.75/0.69	0.74/0.70	0.86/0.81	0.61/0.59	0.37/0.34	0.77/0.71	0.73/0.70	0.67/0.65	0.72/0.55	0.78/0.70	0.77/0.77
HYDRA	0.27/0.61	0.38/0.31	0.38/0.32	0.41/0.33	0.46/0.61	0.29/0.37	0.15/0.14	0.35/0.34	0.39/0.46	0.34/0.47	0.22/0.07	0.31/0.20	0.44/0.72
**Translocations**															
DELLY (PE)	**1.00**/**1.00**	**0.99**/0.97	**1.00**/0.95	**1.00**/0.89	**0.99**/0.98	**1.00**/0.95	**0.95**/0.89	**1.00**/0.94	**1.00**/0.94	**1.00**/0.95	**0.99**/0.72	**1.00**/0.89	**1.00**/**1.00**
DELLY (SR)	0.85/**1.00**	0.95/0.99	**1.00**/**0.99**	**1.00**/0.97	0.98/0.98	0.99/**0.99**	0.91/**0.95**	**1.00**/0.98	0.99/**0.99**	0.99/**0.99**	0.92/**0.97**	0.99/**0.98**	**1.00**/**1.00**
Breakdancer	0.97/**1.00**	0.98/**1.00**	0.99/**0.99**	**1.00**/**0.99**	**0.99**/**1.00**	0.97/0.51	0.61/0.25	**1.00**/**1.00**	0.99/0.98	0.98/0.88	0.94/0.78	0.99/0.97	0.98/**1.00**
GASV	**1.00**/**1.00**	**0.99**/0.97	**1.00**/0.95	0.98/0.77	0.98/0.92	**1.00**/0.94	0.88/0.82	**1.00**/0.93	**1.00**/0.90	0.90/0.59	0.65/0.30	**1.00**/0.89	**1.00**/**1.00**
	*S/PPV*	*S/PPV*	*S/PPV*	*S/PPV*	*S/PPV*	*S/PPV*	*S/PPV*	*S/PPV*	*S/PPV*	*S/PPV*	*S/PPV*	*S/PPV*	*S/PPV*

Reads were simulated under various settings that are detailed in the header of the table. The four variables are coverage, insert size, insert size standard deviation and read length, and only one variable was changed in each experiment. The default values were selected as coverage = 15×, read length = 75 bp, insert size = 300 bp and insert size standard deviation = 30 bp. Each parameter setting was repeated five times and the median of these five values is reported for each method, the best one is in bold. Pindel and HYDRA do not call translocations or reported less than 10% of the simulated events.

### 3.2 Benchmarking DELLY on 1000GP data

DELLY was also used on population-scale sequencing data of the 1000GP (1000 Genomes Project Consortium, 2010). The input alignments used by DELLY were publicly released BAM files generated by the 1000GP using *hg19* as the reference genome. We tested DELLY on 921 illumina sequenced samples from the 1000GP Phase I, which included the 1000GP pilot phase samples, sequenced at low coverage. To assess correlations between the read length and insert size distribution with DELLY's ability to recover SVs, we focused initially on 635 samples, where only one library was sequenced and for which DELLY reported at least one split-read deletion call. The mean insert size of these libraries was 332 bp (range 108–512 bp) with a standard deviation of 33 bp (range 11–120 bp) and a mean read length of 72 bp (range 27–106 bp). There was a significant correlation between the number of split-read deletion calls (e.g. with a median of 442 calls per 1000GP pilot sample) and the read length (Pearson correlation, *c*=0.25; *P* <2.8×10^−10^) as well as the insert size (*c*=0.46,*P* < 2.2×10^−16^). For increasing insert size variability relative to the mean, we observed a significant anti-correlation (*c*=−0.26,*P* < 6.1×10^−11^). These correlation coefficients could be confirmed by a quality-filtered set of paired-end calls (≥3 supporting pairs, avg. mapping quality ≥20) with regard to insert size (*c*=0.24,*P* =7.2×10^−10^) and increasing insert size variability (*c*= −0.19,*P* = 7.5×10^−7^).

**Fig. 6.**
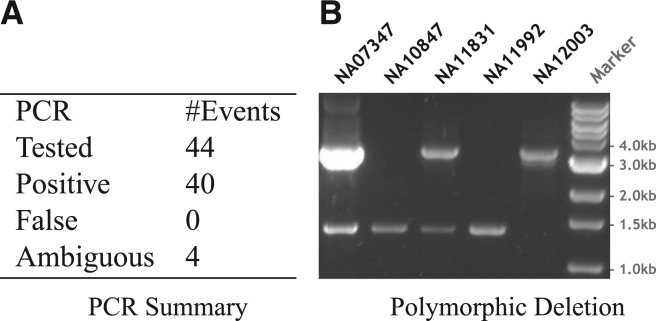
(A) PCR results for 44 randomly selected split-read deletion calls in 5 samples. (B) A polymorphic deletion site (*chr5*:60001704-60003666) that is homozygous alternative in NA10847 and NA11992, heterozygous in NA07347 and NA11831, and homozygous reference in NA12003

We carried out polymerase chain reaction (PCR) validation experiments on five pilot samples (NA07347, NA10847, NA11831, NA11992 and NA12003) to assess the accuracy of SVs discovered by DELLY. Out of 44 randomly selected deletion loci with split-read support, we could unambiguously assign a true-positive outcome to 40 calls (Fig. 6). Four calls were unclear due to a PCR failure or a band outside of the expected SV size range. This gives rise to a conservative estimated PCR-based FDR of ≤9.1% for DELLY, which underlines the high specificity of DELLY (SR) and confirms the results obtained from the simulations. We further compared the DELLY calls to the recently released set of 8384 assembled deletions (lifted from *hg18* to *hg19*) of the 1000GP's SV group pilot project analyses (Mills *et al.*, 2011). While this set was generated by 19 different SV prediction methods, DELLY alone recovered 76% of the joint SV group's calls (using a 90% reciprocal overlap). In the size-range where DELLY operates, i.e. deletions ≥200 bp, DELLY identified 86% of the calls previously released by the 1000GP. Including the DELLY calls from the additional Phase I samples with longer reads, DELLY recovered 86% of all reported deletions and 92% for deletions ≥200 bp, out of which 69% and 82% were supported by split-reads.

### 3.3 Complex genomic rearrangements

**Fig. 7.**
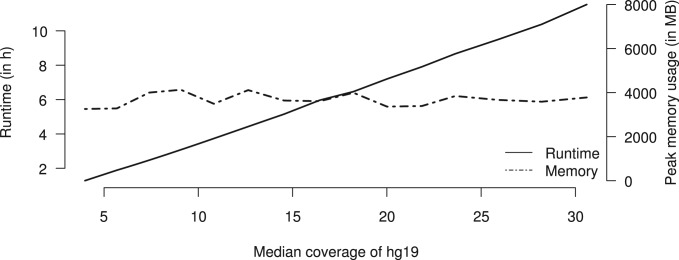
Computational requirements of DELLY for a human resequenced short-read dataset across different coverage levels for deletion discovery

DELLY was also used in a recent study focusing on pediatric brain tumors in the context of the International Cancer Genome Consortium (ICGC) (Hudson *et al.*, 2010; Rausch *et al.*, 2012). DELLY identified a multitude of complex rearrangements, which created ‘deletion-type’, ‘tandem-duplication-type’ or ‘inversion-type’ paired-end mapping signatures but differed from such simple events by means of spanning megabases of sequences and lacking read-depth support. This complex SV landscape could be explained by a set of presumed circular minichromosomes (double minute chromosomes) in which distal genomic segments were rejoined in a seemingly random manner during a single, catastrophic molecular event, termed chromothripsis (Rausch *et al.*, 2012). All tested inter-and intrachromosomal connections on these minichromosomes could be validated by PCR, including 8 interchromosomal, 13 ‘inversion-type’, 3 ‘tandem-duplication-type’ and 3 ‘deletion-type’ connections. Taking these PCR-verified rearrangements as the gold standard set, both DELLY and Breakdancer recovered all rearrangements, whereas Pindel detected none, which may not be surprising since those rearrangements are beyond the size range (1–10 kbp) that Pindel is most suited for. An evaluation of the computational requirements of DELLY using a single CPU (Intel Xeon X5472, 3 GHz) shows that DELLY can be used in routine analyses (Fig. 7), where a further speed-up can be achieved by a naive parallelization on the chromosome level, except for translocations.

## 4 DISCUSSION

The ability to integrate paired-end data from different insert size libraries with split-read data makes DELLY a versatile tool for analyzing SVs in MPS data from various sources, including deep whole-genome sequencing data and low-pass mate-pair sequencing data with longer inserts, with another possible future application area being exome capture data sequenced with paired-ends. Our analyses showed that DELLY (PE) provides excellent sensitivity, whereas DELLY (SR) greatly increases specificity (at nucleotide resolution). Nonetheless, neither DELLY (PE) nor DELLY (SR) alone are optimal across all sequencing parameters. The specificity of DELLY (PE) deteriorates with increasing sequencing coverage due to spurious paired-end calls with low support. The sensitivity of DELLY (SR), however, depends on a sufficient read length and coverage, limitations that call for further research. In particular, population scale sequencing data will enable the genotyping of SVs across cohorts and read-depth distribution modeling the inference of exact copy-number states for unbalanced rearrangements (Handsaker *et al.*, 2011). Third-generation sequencing technologies including nanopore sequencing will additionally facilitate the discovery, and possibly phasing, of SVs in population-scale and cancer genome studies. These types of data should be particularly suited for split-read alignment methods and assembly-based approaches, owing to the increased read length.
